# Nurse work engagement impacts job outcome and nurse-assessed quality of care: model testing with nurse practice environment and nurse work characteristics as predictors

**DOI:** 10.3389/fpsyg.2014.01261

**Published:** 2014-11-13

**Authors:** Peter Van Bogaert, Danny van Heusden, Olaf Timmermans, Erik Franck

**Affiliations:** ^1^Centre for Research and Innovation in Care, Nursing and Midwifery Sciences, University of AntwerpAntwerp, Belgium; ^2^Nursing, Antwerp University HospitalAntwerp, Belgium; ^3^Academy of Health and Welfare, HZ University of Applied SciencesVlissingen, Netherlands; ^4^Department of Health Care, Karel de Grote University CollegeAntwerp, Belgium

**Keywords:** burnout, job satisfaction, nurse retention, nurse practice environment, quality of care, structural equation modeling

## Abstract

**Aim:** To explore the mechanisms through which nurse practice environment dimensions, such as nurse–physician relationship, nurse management at the unit level and hospital management and organizational support, are associated with job outcomes and nurse-assessed quality of care. Mediating variables included nurse work characteristics of workload, social capital, decision latitude, as well as work engagement dimensions of vigor, dedication and absorption.

**Background:** Understanding how to support and guide nurse practice communities in their daily effort to answer complex care most accurate, alongside with the demand of a stable and healthy nurse workforce, is challenging.

**Design:** Cross-sectional survey.

**Method:** Based on earlier empirical findings, a structural equation model, designed with valid measurement instruments, was tested. The study population included registered acute care hospital nurses (*N* = 1201) in eight hospitals across Belgium.

**Results:** Nurse practice environment dimensions predicted nurses’ ratings of job outcome variables as well as quality of care. Features of nurses’ work characteristics, e.g., perceived workload, decision latitude, social capital, and the three dimension of work engagement, played mediating roles between nurse practice environment and outcomes. A revised model, using various fit measures, explained 60% of job outcomes and 47% of nurse-assessed quality of care.

**Conclusion:** The findings in this study show that nurse work characteristics as workload, decision latitude, and social capital, alongside with nurse work engagement (e.g., vigor, dedication, and absorption) influence nurses’ perspective of their nurse practice environment, job outcomes, and quality of care. The results underline aspects to considerate for various stakeholders, such as executives, nurse managers, physicians, and staff nurses, in setting up and organizing health care services.

## INTRODUCTION

Stress and well-being in staff nurses are relevant indicators of nurses’ working conditions, the inter-personal mono- and inter-disciplinary relationships with colleagues, with patients and the quality of care nurses provide. Staff nurses often work in problematic practice environments, characterized with various difficulties and stress-factors that can undermine staff nurses’ full capacity to provide excellent care. International insights and empirical studies show the importance of balanced, healthy and supportive nurse practice environments and psychosocial work environments to achieve and sustain stable and high performance nurse workforces (Rafferty et al., 2001; Estabrooks et al., 2002; Choi et al., 2004; Vahey et al., 2004; Gunnarsdóttir et al., 2007; Li et al., 2007; Leiter and Maslach, 2009; Schubert et al., 2009; Kowalski et al., 2010). These types of nurse practice environments are characterized by high levels of job satisfaction and engagement, relatively low levels of stress, burnout and turnover rates, as well as favorable scores on quality of care and patient safety indicators as mortality, co-morbidity, and serious adverse events (Tourangeau et al., 2005; Laschinger and Leiter, 2006; Aiken et al., 2008; Friese et al., 2008). The challenge for healthcare organizations, such as acute care hospitals, is to enhance and sustain nurse practice environments that maximize healthcare workers capacities, wherein staff nurses provide the best care answering complex patients needs. To set up, organize, and sustain supportive nurse practice environments is complex and can be undermined through various paradoxical concerns, matters and goals between top-level management, physicians, staff nurses, and nursing teams.

Previously, our research team investigated the relationships between nurse practice environment, job outcomes, and nurse-assessed quality of care through nurse work characteristics (e.g., workload, decision latitude, and social capital) and feelings of burnout (e.g., emotional exhaustion, depersonalization, and personal accomplishment; Van Bogaert et al., 2013a). These relationships were tested using structural equation modeling. Feelings of burnout (when relatively mild or low) were modeled as mediating outcome variables that impacted dependent outcome variables of job outcomes (e.g., relatively high job satisfaction, less intention to leave the nursing profession or the hospital) and favored nurse-assessed quality of care (at the unit, the last shift, and the hospital). In the confirmed model, the independent variables nurse practice environment, through nurse–physician relationship, nurse management at the unit level and hospital management and organizational support, when favorable assessed by staff nurses, predicted positive scores on job outcome variables (e.g., relatively low feelings of burnout, job satisfaction, less intention to leave the nursing profession and the hospital and favorable nurse-assessed quality of care). This model was systematically developed and tested in various stages and study populations (e.g., acute care hospital nurses and psychiatric care hospital nurses; Van Bogaert et al., 2009b, 2013b).

Leiter and Spence Laschinger (2006) showed impact of nurse practice environment aspects such as leadership, nurse–physician relationship, policy development, nursing staffing and nursing model of care on burnout dimensions (e.g., emotional exhaustion, depersonalization, and personal accomplishment), described as the Nursing Worklife Model. Moreover, the Nursing Worklife Model was extended in a following study with an impact of nurse practice environment aspects on patient adverse events through feelings of burnout (Laschinger and Leiter, 2006). A mediating position of burnout was also confirmed by a study of Leiter and Maslach (2009), performed with a nurse population between six areas of worklife, described as keys to person–job fit (e.g., the extend of perceived workload, control, reward, fairness, community, and shared valued) and turnover intentions. In addition, research showed associations linkages of nurse-reported workload as well as decision latitude and social capital with emotional exhaustion (Kowalski et al., 2010). Our premier study results empirically demonstrated that social capital and decision latitude supported by nurse practice environments influenced outcome variables such as burnout, job outcomes, and nurse-assessed quality of care.

Simultaneously, a comparable model was tested with work engagement defined by vigor, dedication, and absorption instead of burnout variables, using a psychiatric hospital care nurse population (Van Bogaert et al., 2013c). In the model, work engagement was defined as a positive affective motivational state of fulfillment, manifested as vigor, dedication, and absorption, and could be recognized as an independent, distinct (albeit related) concept that is negative related to burnout (Schaufeli and Bakker, 2003). Moreover, Maslach and Leiter (2008) considered the psychological relationships of workers to their jobs as a continuum between negative experiences of burnout and the positive experiences of engagement. They describe three dimensions with contrasting poles: exhaustion versus energy, cynicism versus involvement, and inefficacy versus efficacy. Maslach (2011) argues that burnout was developed from a grassroots, bottom–up, qualitative approach in which people were asked to describe their work experiences. In contrast, the author noticed that work engagement was originally defined from a theoretical perspective – either as the opposite of burnout or as an independently positive state. To characterize person–job fit work engagement (the positive one) has been recently studied by researchers instead of burnout (the negative pole) that has been widely studied (Maslach and Leiter, 1997, 2008). Moreover, examining work engagement is consistent with proactive support of positive job experiences rather than identifying negative person–job fits once they have arisen (Leiter and Bakker, 2010). In a longitudinal study design with a large population of health employees (*n* = 3.110) Armon et al. (2012) found that changes in the levels of job demands, job control, and social support over time predicted subsequent certain changes in levels of vigor over time. The growth of interest in work engagement is potentially a reflection of widespread recognition that is making effective use of employee skills and knowledge with proper support and resources and is imperative in rapidly changing economies and organizations (Kanter, 1993; Leiter and Bakker, 2010; Van Bogaert et al., 2013a). Previous empirical studies showed that nurses perceptions of sufficient support (e.g., peers and supervisors) and sufficient resources needed to do the job, in accordance with opportunities to be involved in joint-decision making, are linked with job satisfaction, commitment, engagement, productivity, and quality of care (Laschinger et al., 2004, 2009; Laschinger and Finegan, 2005). Our study results have shown that work engagement is a likely direct consequence of practice environments that may ultimately have impacts on both staff and patient outcomes.

**FIGURE 1:**
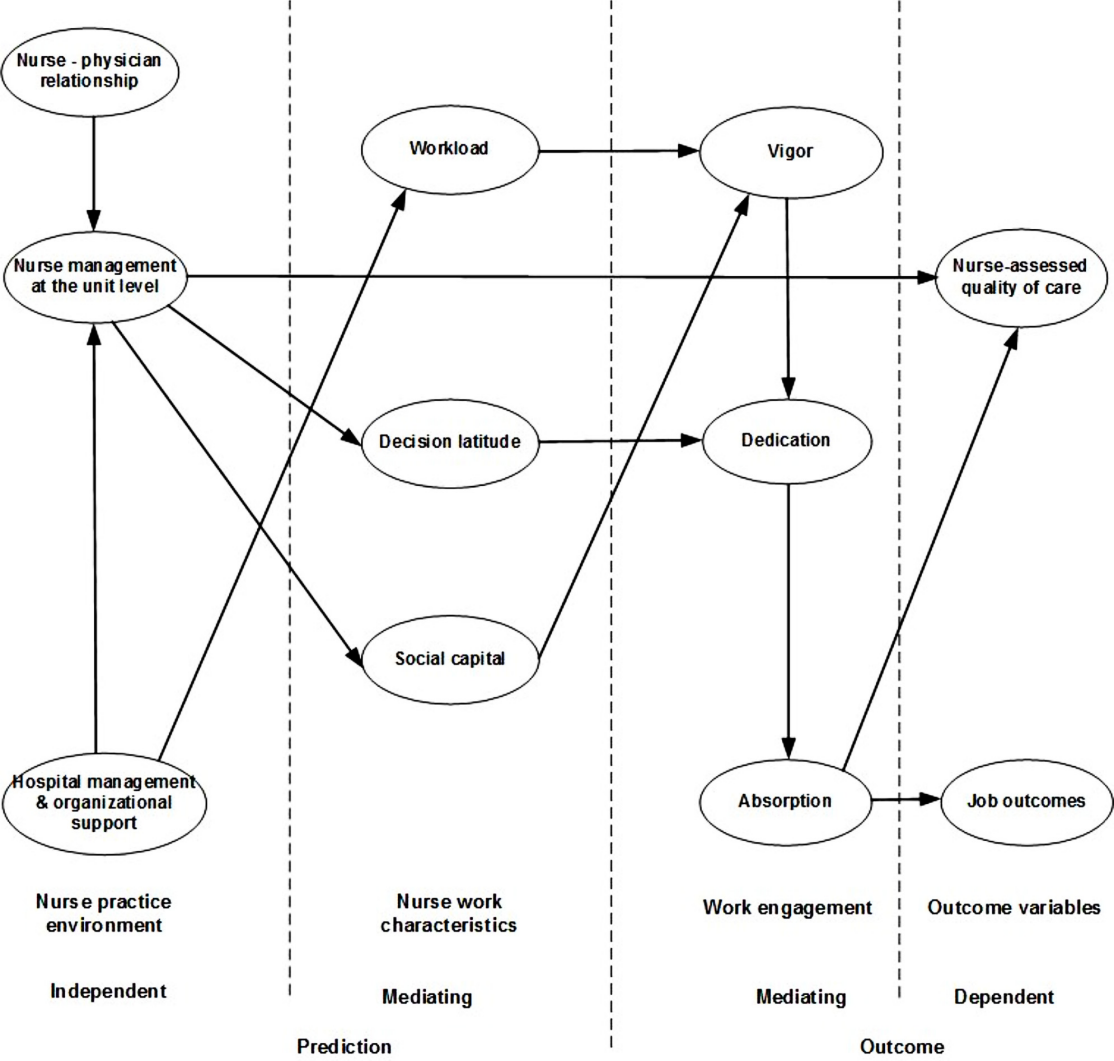
**Tested model**.

The aim of this study was to investigate the relationships between nurse practice environment variables and the outcome variables job outcomes and nurse-assessed quality of care, using structural equation modeling. The relationships were tested with nurse work characteristics as mediating predictors and work engagement as mediating outcome variables (see **Figure 1**). In the tested model we hypothesized that vigor has an impact on both outcome variables (e.g., job outcomes and nurse-assessed quality of care) through dedication and absorption (Van Bogaert et al., 2013c). As seen in our previous tested model (Van Bogaert et al., 2013c) hospital management has an impact on vigor through workload. We expect high scores on vigor if hospital management supports nurses to control their work demands; otherwise we expect lower scores when nurses experience difficulties to balance their work demands. When nurse management at the unit level, supported by physicians and hospital management, sufficiently involves nurses in (clinically as well as organizationally) decision-making processes (decision latitude) and supports team cohesion and collaboration (social capital), scores on dedication and vigor will be more favorable. Moreover, nurses who are engaged through high score of vigor and dedication will be more focused (absorption) with their daily tasks (Van Bogaert et al., 2013c). Nurse management at the unit level has also direct impact on nurse-assessed quality of care (Van Bogaert et al., 2010, 2013c, 2014).

## MATERIALS AND METHODS

### STUDY POPULATION

The study had a cross-sectional design conducted in two hospitals (one 700-bed general hospital and one 600-bed university hospital) in the Dutch speaking part of Belgium, as well as in one hospital group with six hospitals (number of beds ranged from 125 to 320) in the French speaking part of Belgium. All participants were professional (registered) nurses working in direct care in medical, surgical, and intensive care units and operating theaters, or adult or pediatric care units. Participants were invited by a coordinator/contact person at each institution to voluntarily complete questionnaires; data collection took place between June 2011 and June 2012. In total, 1201 professional (registered) nurses completed the questionnaire.

### MEASUREMENT INSTRUMENTS

*Practice environment* was measured with translated and validated Dutch and French versions of the *Revised Nursing Work Index (NWI-R),* adapted for the Belgian context (Aiken and Patrician, 2000). Three dimensions or subscales have been identified in the Belgium version of the NWI-R (Van Bogaert et al., 2009a): nurse–physician relations (three items), nurse management at the unit level (13 items), and hospital management and organizational support (15 items). Staff nurses rated their agreement with various statements regarding the practice environment in their current positions on a 4-point Likert-type scale (*strongly disagree, disagree, agree, strongly agree*). *Work Engagement* was measured with Utrecht Work Engagement Scale (*UWES)*, a nine-item short version measure (Schaufeli and Bakker, 2004; Schaufeli et al., 2006; Van Bogaert et al., 2013c) tapping three separate dimensions with each three items; vigor, dedication, and absorption. *Vigor* is defined as high levels of energy and mental resilience at work. *Dedication* is described as strong involvement in one’s work accompanied by feelings of enthusiasm and significance. *Absorption* relates to being fully engrossed in one’s work and having difficulties detaching oneself from it. Respondents rated the frequencies with which they experienced various job-related feelings on a 7-point scale ranging from *never to every day*. Schaufeli and Bakker (2010) concluded that work engagement assessed by the UWES is a unitary construct that is constituted by three different yet closely related dimensions. The three-factor structure appeared stable across study populations from different countries and occupational groups within slightly difference in values of factor loadings and correlations. In addition, the short version was found stable over time.

*Nurse work characteristics* (Van Bogaert et al., 2013a) were measured based on three measurement scales; workload, decision latitude, and social capital. *Workload* was measured with the Intensity of Labor Scale of Richter et al. (2000) included six statements whereon respondents rated their agreement or disagreement with on 4-point Likert-type scales (*strongly disagree, disagree, agree, strongly agree)*. *Decision latitude* (Richter et al., 2000) was measured using a seven-item measurement instrument, whereby respondents were asked their agreement on their ability to make decisions, be creative, and use and develop their professional and personal skills at the workplace. Respondents rate each item on a 4-point Likert-type scale (*strongly disagree, disagree, agree, strongly agree)*. *Social capital* was measured with a six-items rated scale, asking respondents their agreement on 4-point Likert-type scale (*strongly disagree, disagree, agree, strongly agree)* on shared values and perceived mutual trust within teams and organizations (Pfaff et al., 2004; Ernstmann et al., 2009).

To measure the *Nurse – assessed quality of care,* nurses were asked to rate their perceived quality of care overall on their units, on the last shift, and in the hospital over the last year on a 4-point Likert-type scales (*poor, fair, good, excellent*). Finally, three types of *job outcomes* were assessed: satisfaction with the current job (*very dissatisfied, dissatisfied, satisfied, very satisfied*), intention to leave the hospital within the next year (yes, no), and intention to leave the nursing profession (*yes, no*).

The structures of multi-item measures were thoroughly evaluated with exploratory and confirmatory factor analysis and internal consistency analysis in several previous samples (Van Bogaert et al., 2009a,b, 2013b,c) and current sample (Van Bogaert et al., 2013a). The confirmation of the three-factor structure of both the NWI-R and UWES, as well as the one-factor structure of workload, was based on various fit measures with previous and current study population. The confirmation of decision latitude and social capital were based on various fit measures with the current study population. Sufficient model fit were tested with Comparative Fit Index (CFI > 0.90), Incremental Fit Index (IFI > 0.90), and Root Square Error of Approximation (RMSEA < 0.08; Van Bogaert et al., 2009b, 2013a,b,c).

All multi-item scales have Cronbach’s alpha coefficients ranged from 0.65 to 0.90, except the job outcome dimension (0.32). As identified with previous and current study populations, the inter-item correlations (an alternative measurement technique assessing internal consistency; Briggs and Creek, 1986) for the indicators of the job outcome dimension ranged from fair to moderate with values between 0.15 – 0.21.

All variables, with the exception of workload, were coded for analysis whereby higher scores indicated a stronger agreement or more favorable ratings. On the latter measure, higher scores are suggestive of unfavorable perceptions or conditions.

### DATA ANALYSIS AND MODEL TESTING

Preparing for model testing, the data were analyzed descriptively and correlations were computed. The Statistical Package for the Social Science (SPSS) version 22.0 and AMOS version 22.0 software (SPSS Inc, Chicago, IL, USA) were used for descriptive analyses and computation of Cronbach’s alphas and correlation coefficients, and model testing by structural equation modelling (SEM).

In SEM, a ratio of at least five subjects for each variable, including error measurements, observed variables (indicators), and latent variables (dimensions), is recommended (Bentler and Chou, 1987). Based on our earlier work (Van Bogaert et al., 2009b, 2013a,b,c), a content-driven selection of observed variables (see **Table 1**) was made to equalize measure weighting across indicators (Byrne, 1994, 2001, 2010). For example, the nurse management at the unit level scale included a selection of items related to the nurse manager, the clinical competence of colleagues and the availability of nursing care plans, as well as standardized policies and procedures. A total of 85 variables (error measurements, observed and latent variables) were included in the model and analyzed with a sample of 1,201 respondents. AMOS software was used to conduct model testing on the full database incorporating imputation of incomplete data, maximum likelihood estimation, and estimation of means and intercepts (Arbuckle, 2005). To verify and improve model plausibility, various fit measures were calculated and compared against accepted criterion levels (CFI and IFI ≥ 0.90; RMSEA < 0.080). To achieve optimal model fit, assessed using standard measures, pathways were included or trimmed based on the impacts on chi-square statistics through modification indices, as well as on empirical and theoretical grounds. In addition, not statistically significant pathways were deleted. To determine whether or not to include additional parameters in the model, Byrne (2010) highlight the prime importance of the extent to which they are substantively meaningful and the model exhibits adequate fit.

**Table 1:** Observed (a) and latent variables (b) of the improved model (*n* = 1.201).

		Loading
**Nurse practice environment**
Nurse-physician relationship (b)	
2	Physicians and nurses have good working relationships (a).	0.70
27	Much teamwork between nurses and doctors (a).	0.79
39	Collaboration (joint practice) between nurses and physicians (a).	0.86
Nurse management at the unit level (b)		
33	Working with nurses who are clinically competent (a).	0.47
44	Nurse managers consult with staff on daily problems and procedures (a).	0.45
51	Standardized policies, procedures and ways of doing things (a).	0.37
Hospital management and organizational support (b)	
14	A chief nursing officer is highly visible and accessible to staff (a).	0.61
36	An administration that listens and responds to employee concerns (a).	0.85
38	Staff nurses are involved in the internal governance of the hospital (e.g., practice and policy committees (a).	0.56
**Nurse characteristics**
Workload (b)
4	Many times I have to do a lot of work (a).	0.67
7	Tasks that I have to solve are often very difficult (a).	0.88
13	Normally time is short, so often I am pressed for time at work (a).	0.68
Decision latitude (b)
3	In my work I can participate in new developments (a).	0.51
10	I can organize my work independently (a).	0.46
12	In my work I have to take a lot of decisions independently (a).	0.29
Social capital (b):	
2	In our unit there is trust between nurses (a).	0.75
4	In our unit there is favorable work climate (a).	0.79
6	In our unit nurses shared values (a).	0.74
**Burnout**
Vigor (b)	
2	At my job, I feel strong and vigorous (a).	0.72
5	When I get up in the morning, I feel like going to work (a).	0.83
Dedication (b):	
3	I am enthusiastic about my job (a).	0.89
4	My job inspires me (a).	0.83
Absorption (b)	
8	I am immersed in my work (a).	0.68
9	I get carried away when I’m working (a).	0.71
**Outcome variables**
Job outcomes: (b)	
1	Job satisfaction (a).	0.59
2	Intention to stay in the hospital (a).	0.34
3	Intention to stay in nursing (a).	0.32
Nurse – assessed quality of care (b)	
1	At the current unit (a).	0.87
2	At the last shift (a).	0.70
3	In the hospital the last year (a).	0.47

## RESULTS

Response rates at the hospital level ranged from 44 to 74% with a total study sample of 1201 (*N* = 244 general hospital, *N* = 440 university hospital, and *N* = 517 hospital group). The final sample was 57% Dutch speaking and 43% French speaking.

**FIGURE 2:**
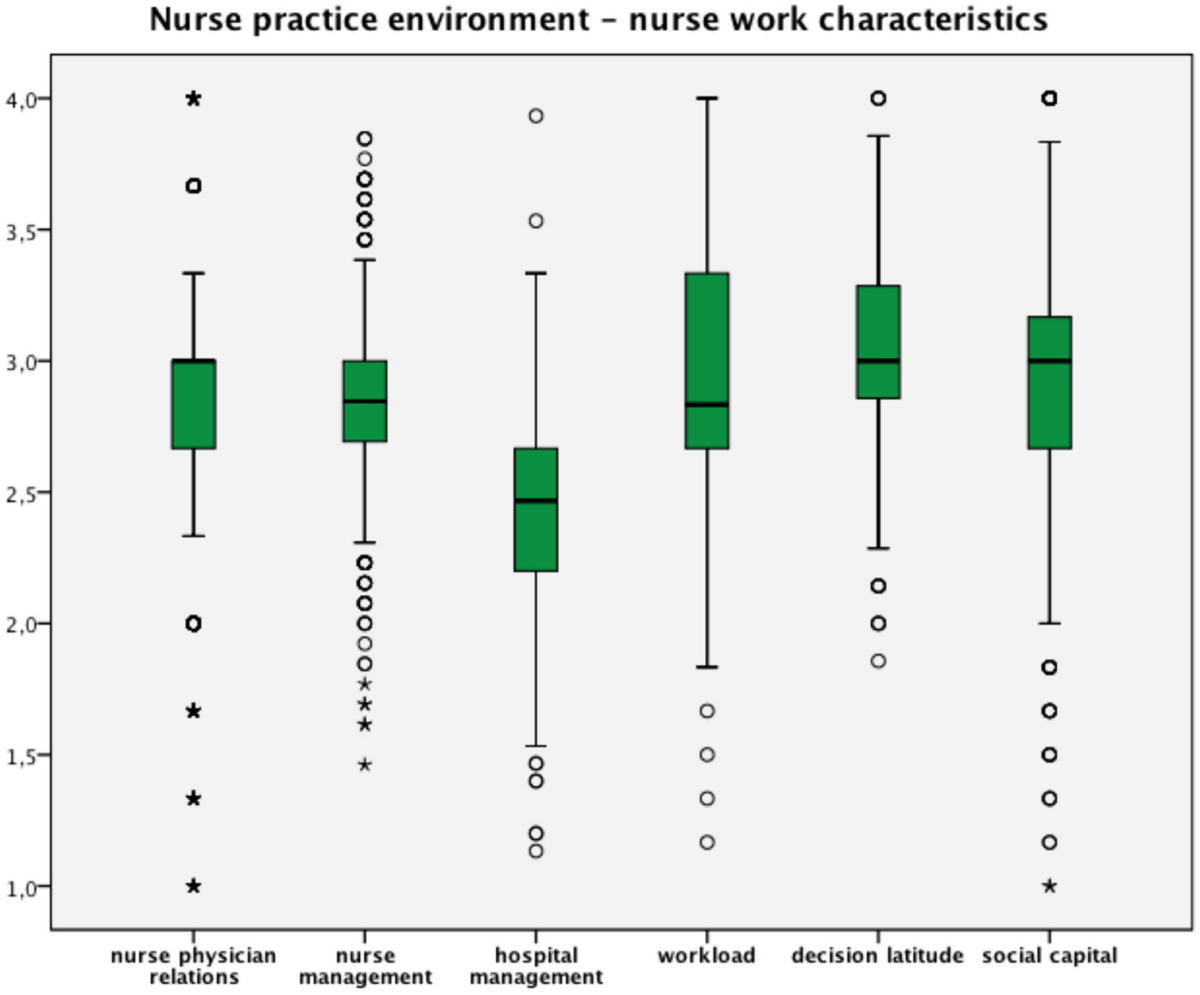
**Nurse practice environment and nurse work characteristics.** Scale range 1–4; all scales higher values means favorable ratings expect for workload; 2.5 is the midpoint and means neither favorable nor unfavorable.

**Table 2** shows study populations’ demographic variables and the distribution of outcome variables. Results show less than 10% of the staff nurses were dissatisfied or very dissatisfied, where almost 6 and 11% had the intention to leave the hospital last year and the nursing profession. Respectively almost 13 and 10% assessed the quality of care at the unit and the last shift fair or poor and almost two out of five nurses assessed the quality in the hospital the last year as deteriorated or definitely deteriorated. Nurse–physician relations, nurse management at the unit level, decision latitude and social capital were rated favorably (scores of > 2.5 reflect predominantly positive responses to questions about desirable elements being present). The average respondents, however, rated hospital management and organizational support (<2.5) and workload (>2.5) unfavorable (see boxplots **Figure 2**). Based on cut-off values defined by Schaufeli and Bakker (2003), respectively, 45, 62, and 52% had high or very high levels of vigor, dedication, and absorption.

**Table 2:** Characteristics of nurses and distribution of nurse job outcomes and nurse-reported quality of care (*n* = 1.201).

Nurse characteristics	Mean	SD
Age in years	38.3	10.3
Years in nursing	15.3	10.3
Years on present unit	9.5	8.8
	***N***	**%**
Female	1.023	85.2
Baccalaureate degree in nursing	919	76.5
Master degree in nursing	21	1.8
Working regime 50 % or less of a full-time position	358	29.8
Working regime 75 % or more of a full-time position	722	60.1
**Outcome variables**	***N***	**%**
Dissatisfied or very dissatisfied with the current job	100	8.3
Intention to leave the current hospital within one year	71	5.9
Intention to leave nursing	131	10.9
The quality of care on the unit is fair or poor	154	12.8
The quality of care at the last shift is fair or poor	113	9.4
The quality of care in hospital the last year has deteriorated or definitely deteriorated	114	39.5

The correlation matrix (**Table 3**) shows all variables were predominantly significant positive correlated except for all correlations with workload.

**Table 3:** Correlation analysis between studied variables.

		Mean–SD	2	3	4	5	6	7	8	9	10	11
1	Nurse–physician relations	2.83-0.53	0.313**	0.358**	0.251**	-0.048	0.252**	0.185***	0.164***	0.168***	0.231**	0.115**
2	Nurse management	2.87-0.33		0.490**	0.320**	-0.146**	0.483**	0.235***	0.266***	0.243***	0.434**	0.331**
3	Hospital management	2.43-0.36			0.262**	-0.260**	0.301**	0.314***	0.307***	0.289***	0.381**	0.312**
4	Decision latitude	3.01-0.33				0.223**	0.285**	-0.207***	0.270***	0.281***	0.217**	0.164**
5	Workload	2.95-0.51					-0.068*	-0.200**	-0.075*	-0.60*	-0.162**	-0.193**
6	Social capital	2.95-0.52						-0.251**	0.236***	0.220*	0.377**	0.262**
7	Vigor #	4.31–1.2							0.686***	0.667***	0.269***	0.355***
8	Dedication #	4.85–1.1								0.755***	0.306**	0.398**
9	Absorption #	4.11-1.4									0.283***	0.310***
10	Nurse-assessed quality of care	2.90-0.48										0.290**
11	Job outcomes ##	1.79-0.20										

*p < 0.05; **p < 0.01; ***p < 0.001; Mean–SD of scales calculated with all items as described in the Section “Materials and Methods”; scale range 1–4 except # range 0–6, ## range 1–2.

**FIGURE 3:**
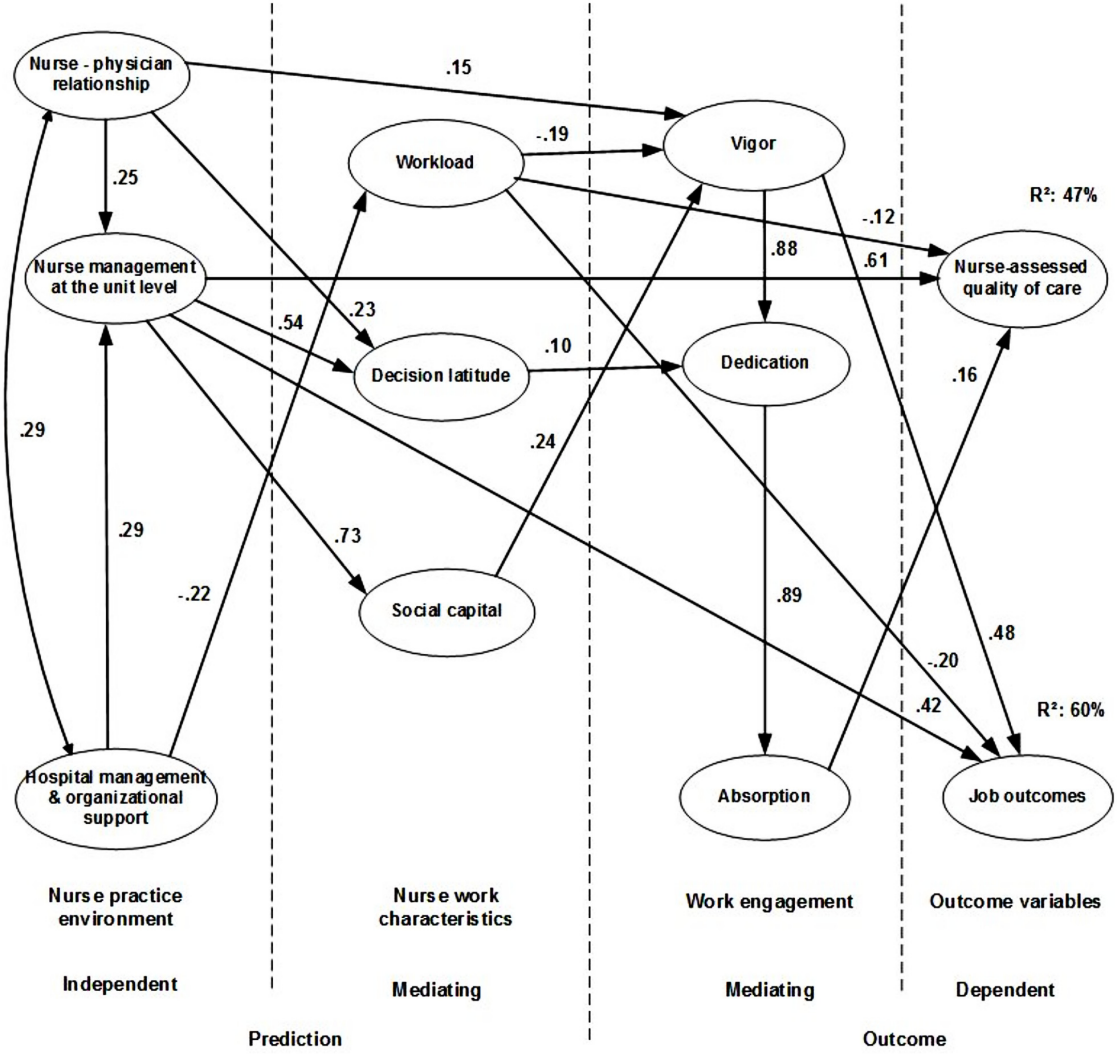
**Improved model**.

Fit measures of the hypothesized model were insufficient (CFI: 0.887; IFI: 0.888) and modifications as described in the method section were necessary. Deletion and inclusion of pathways and in addition the deletion of item 1, 7, and 6 from respectively, work engagement dimensions vigor, dedication, and absorption (e.g., modification indices between item error measurements within vigor and dedication) gave sufficient fit measure and confirmed an adjusted model (CFI: 0.908; IFI: 0.907; RMSEA: 0.048). The improved model (**Figure 3**) showed additional pathways between nurse–physician relationship and respectively, vigor and decision latitude, nurse management at the unit level and job outcome, and workload and respectively, nurse-assessed quality of care and job outcome. Pathways between hospital management and organizational support and absorption and between the latter and job outcome were deleted. The improved model explained 47% of the variances on nurse-assessed quality of care and 60% of the variance on job outcome. Nurse management at the unit level has a strong direct impact on outcome variables with explained variances of 22 and 36%, respectively.

## DISCUSSION

A previously tested model showed the impact of burnout dimensions emotional exhaustion, depersonalization, and personal accomplishment on job outcomes and nurses-assessed quality of care, predicted by nurse practice environment and nurse work characteristics (Van Bogaert et al., 2013a). In the current study, we confirmed a model with work engagement dimensions that impacts job outcomes and nurse-assessed quality of care, tested with the same predictors. In both models, favorable nurse management at the unit level had a strong direct positive effect on both dependent outcome variables. In addition, hospital management had a direct effect on workload and, in turn workload had a direct negative effect on both dependent outcome variables, less strong, although, than nurse management at the unit level. As hypothesized, favorable nurse management at the unit level had a direct positive effect on decision latitude and social capital and both variables, in turn have a direct positive effect on dedication and vigor. Additionally, nurse–physician relationship had a direct positive effect on vigor and vigor had a direct positive effect on job outcomes and dedication and indirect on absorption and, in turn the latter had a direct positive effect on nurse-assessed quality of care. The variance of nurse-assessed quality of care (at the unit, the last shift, and the hospital last year) and job outcomes (job satisfaction, intention to leave the nursing profession and the hospital last year) explained by the improved model were 47 and 60%, respectively.

The study results suggest the importance to align various concerns, issues, and goals between top-level management, physicians and nurse management, to create supportive practice environments that balance workloads and provide sufficient autonomy for nurses through decision latitude and with attention for interpersonal relationships within the nursing teams (e.g., social capital). These conditions stimulate work engagement and were associated with job satisfaction, lower turnover rates as well favorable nurse-assessed quality of care at the unit, the last experienced shift and in the hospital over the last year. The latter is indicative for supportive collaborations within hospital teams and departments. Indirectly, but not demonstrated in this study, we suggest that low levels of feelings of burnout and high levels of engagement, predicted by favorable work conditions as found in our study results, also will be supportive for staff nurses perceived general health (e.g., stress, fatigue, emotional drained, mild physical complaints such as headaches, nausea, dyspepsia, sleep disturbance) and low levels of absenteeism. Shirom et al. (2008) found that the affective state of vigor and objectively assessed functional capacity interact to predict subsequent changes in self-rated health. A recent systematic review found that high levels of work related stress, burnout, job dissatisfaction, and poor health are common within the nursing profession (Khamisa et al., 2013). In addition, the authors remark that nurses experience longer working hours, as well as frequent direct, personal, and emotional contact with a large number of patients in comparison with other health professionals. In our study, perceived workload had a prominent mediating and direct negative effect on both outcome variables. Besides the necessary attention for the more *soft* nurse work characteristics as decision latitude and social capital through the empowerment of nurses and team cohesion (Laschinger and Finegan, 2005; Kowalski et al., 2010) more insight and knowledge of the *hard* nurse work characteristic, e.g., workload, seems essential. A longitudinal study of our research group (Van Bogaert et al., 2014) in one hospital found positive effects on similar outcome variables through a hospital transformation process from classic hierarchical and departmental organization into a flat and interdisciplinary. In addition, to create better care environments and outcomes, the implementation of the *Productive Ward – Releasing Time to Care*^TM^ *program* within the hospital strategy was ongoing and showed additional positive effects on study variables. Otherwise, research on cognitive and physical workloads and work demands of staff nurses can guide interventions to improve care environments, achieving more general health of the nursing workforce, as well as better quality and safety of care (Hoonakker et al., 2011; Kurowski et al., 2014). Moreover, studies indicate that adequate staffing levels and proper qualifications of staff nurses are also associated with better nurse outcomes as well as patient outcomes (Aiken et al., 2014). Therefore, executives, physicians, nurse managers as well as staff nurses share responsibility to tackle also issues around workload due to the negative effect on well-being by the risk to threaten the positive pole of engagement (e.g., vigor; e.g., improved model) and the risk to strengthen the negative pool of burnout (e.g., emotional exhaustion; Van Bogaert et al., 2013a) and the negative effect on described outcomes.

As Kanter (1993) described the value of structural empowerment in organizations to cope adequately with changes and evolving needs of markets and customers, it will be important for healthcare organizations such as acute care hospital to support structurally interdisciplinary care delivery settings. Empowerment means enough and structurally access to information, support of subordinates, peers as well as supervisors and opportunities to learn and develop of healthcare workers aiming an excellent patient care answering complex needs. Nurse work characteristics such as balanced workload controlled by nurses themselves, decision latitude through joint-decision making and social capital through shared values and collaboration between healthcare workers are aspects of perceived empowerment with positive impact on outcomes as confirmed in our study. Principles of Magnet Hospital are being used to guide a transformation of the nursing organization to create healthy nurse practice environment conducive to nurse professionalism, retention, productivity, satisfaction, and safe quality patient care. This concept has a strong focus on nurse and patient outcomes within nursing teams, structural empowerment by hospital management, and positive interdisciplinary relations guided by previous empirical studies over more than 30 years (McClure et al., 2002; Kramer and Schmalenberg, 2004; Chen and Johantgen, 2010). The American Nurses Credentialing Center [ANCC] (2005) organizes since more then 20 years Magnet Hospital recognition program in the US and internationally. The program was originally based on *14 Forces of Magnetism* (American Nurses Credentialing Center [ANCC], 2005) and in 2008 transformed the framework to four components: transformational leadership; structural empowerment; exemplary professional practice; and new knowledge, innovations, and improvements (Wolf et al., 2008; American Nurses Credentialing Center [ANCC], 2014). Various studies confirmed favorable outcomes of hospitals organized on Magnet Hospital principles or recognized Magnet hospitals (Laschinger et al., 2003; Lacey et al., 2007; Houston et al., 2012; Kalisch and Lee, 2012; Tinkham, 2013; Van Bogaert et al., 2014). Various variables predict nurse workforce outcomes such as stress and well-being, as well as patient outcomes as hospital mortality. This study results enlightened mechanisms through which nurse practice environment dimensions were associated with job outcomes and nurse-assessed quality of care, identifying mediating variables of nurse work characteristics (e.g., workload, decision latitude, and social capital) and work engagement dimensions (e.g., vigor, dedication, and absorption).

### LIMITATIONS AND FUTURE STUDIES

The study was based on nurses’ self report data and should be interpreted with caution. Because of the cross-sectional design, the confirmed model describes no causality. Replication of the study with various study populations (e.g., within different cultures and health care organizations) is necessary to verify how robust the models’ associations are. Moreover, the *NWI-R 3-factor structure* (nurse–physician relationship, nurse management at the unit level, hospital management and organizational support) is at the moment only confirmed with Belgian study populations. Replication in different socio-economic conditions is necessary to demonstrate generalizability. The study method and used measurements instruments can guide relevant interventions initiatives to improve staff nurses’ practice environment to achieve excellent care and a stable nurse workforce and will extend confirmation of our study results. Nurse-perceived health variables as well as objective variables measuring quality and patient safety will have added value within our study design.

## CONCLUSION

Study findings underline aspects – such as nurse work characteristics (e.g., workload, decision latitude, and social capital) along with nurse work engagement (e.g., vigor, dedication, and absorption) – to considerate for various stakeholders, in setting up and organizing health care services. Alignment of various concerns, issues, and goals between top-level management, physicians, nurse management, staff nurses and, last but not least, patients will offer the capacity to improve health and healthcare.

## Conflict of Interest Statement

The authors declare that the research was conducted in the absence of any commercial or financial relationships that could be construed as a potential conflict of interest.
